# Methane Production of *Sargassum* spp. Biomass from the Mexican Caribbean: Solid–Liquid Separation and Component Distribution

**DOI:** 10.3390/ijerph20010219

**Published:** 2022-12-23

**Authors:** Enrique Salgado-Hernández, Ángel Isauro Ortiz-Ceballos, Sergio Martínez-Hernández, Erik Samuel Rosas-Mendoza, Ana Elena Dorantes-Acosta, Andrea Alvarado-Vallejo, Alejandro Alvarado-Lassman

**Affiliations:** 1 Instituto de Biotecnología y Ecología Aplicada (INBIOTECA), Universidad Veracruzana, Xalapa 91090, Mexico; 2 CONACYT-Tecnológico Nacional de México/Instituto Tecnológico de Orizaba, Av. Oriente 9, 852. Col. Emiliano Zapata, Orizaba 94320, Mexico; 3 División de Estudios de Posgrado e Investigación, Tecnológico Nacional de México/Instituto Tecnológico de Orizaba, Orizaba 94320, Mexico

**Keywords:** *Sargassum*, Caribbean, seaweed, anaerobic digestion, biogas, biochemical methane potential

## Abstract

In the last decade, *Sargassum* spp. seaweed species have caused massive flooding on the Caribbean Sea coasts. These seaweed species have a high content of recalcitrant compounds, such as insoluble fibers and polyphenols, which generate low methane yields in anaerobic digestion (AD). This study investigated the effect of solid–liquid separation of *Sargassum* biomass on biodegradability and methane yield. A biochemical methane potential (BMP) test was conducted with both fractions and raw biomass (RB). A mass balance was developed to assess the distribution of the components. The obtained liquid fraction (LF) showed high biodegradability and a high methane production rate, and it generated a methane yield of 159.7 ± 7.1 N L kg VS^−1^, a value that corresponds to approximately twice that achieved with RB and the solid fraction (SF). The component distribution analysis showed that about 90% of total solids (TS), volatile solids (VS), ash, carbon, and cellulose were retained in the SF. In conclusion, the LF had high biodegradability and methane yield. This suggests the potential for LFs of *Sargassum* biomass to be treated in large-scale high-load reactors; however, studies applied to SFs are needed because they retain a large amount of organic matter with low biodegradability.

## 1. Introduction

Pelagic *Sargassum* consists of two species: *Sargassum fluitans* (Boergesen) and *S. natans* (Linnaeus), which are abundant in the Sargasso Sea located in the northern Atlantic Ocean [1,2]. The Sargasso Sea serves as a spawning and nursery area for many ecologically and commercially important organisms [3]. However, since 2011, a recurring seaweed bloom was observed in satellite imagery in the central Atlantic, often extending from West Africa to Brazil and the Caribbean Sea [4]. In subsequent years, this new location has resulted in large quantities of *Sargassum* spp. appearing in the central Atlantic Ocean and the Caribbean Sea. This causes frequent flooding of the Caribbean coasts and the west coast of Africa, generating serious environmental, ecological, and economic problems [2,5]. The costs for the removal of these seaweed species from beaches in 2018 were estimated at US $210 million, causing major economic impacts [5]. Additionally, the cleanup has not covered the entire coastline because 90% of the coastline is not considered of importance to tourism; thus, thousands of tons of algae accumulate annually along the coast [2,6]. The algae that manage to be extracted from the beach and ocean are disposed of in areas that are not adequately prepared to prevent leachates from entering aquifers [2].

Diverse studies have proposed several sustainable applications for *Sargassum* biomass, including (1) use in agriculture as fertilizer, (2) marine- and industrial-effluent bioremediation based on biosorption of heavy metals, (3) food products or ingredients, (4) obtaining biomolecules with use in the pharmaceutical industry, and finally, (5) bioenergy generation (fuels, energy, and heat) [7,8]. The latter has some disadvantages since some processes require dry feedstock. It has been reported that the energy required to dry seaweed with moisture content >88% can exceed the amount of energy in the dried seaweed [9]. Therefore, methods in which wet biomass can be used directly to produce biofuels can address moisture limitations, and anaerobic digestion (AD) is a good option [8].

AD can convert several brown algae to biomethane with yields ranging from 204 to 380 N L CH_4_ kg VS^−1^ [10]. These values are similar to those achieved with other 1st and 2nd-generation biomasses [11]. However, in the case of AD of pelagic *Sargassum*, yields range from 41 to 145 N L CH_4_ kg VS^−1^ [12,13,14]. Milledge et al. [12] found that the mixture of *Sargassum* species did not generate methane; it was generated only by separating the mixture by species. They reported that high amounts of insoluble fibers and polyphenols influenced the low methane yields. In addition, Tapia-Tussell et al. [13] described that the low methane yields were due to the high content of lignin, phenols, and macrominerals such as Na, K, and Mg present in *Sargassum* spp. biomass.

Insoluble fibers are difficult to degrade, and polyphenols are potential inhibitors in anaerobic digestion [15]. *Sargassum* spp. has been characterized by a high content of insoluble fibers [16] in addition to high concentrations of polyphenols [12,13]. The presence of a high content of insoluble fibers makes seaweed have a lower amount of volatile solids (VS) available, mainly polysaccharides and proteins, for the microorganisms involved in AD [16]. To improve methane recovery from wastes with high solid content, mainly fibers, some authors have employed physical separation of the material in several fractions (solid–liquid) by using screens or mechanical separators [17,18,19]. Separation processes into solid and liquid fractions have been applied mainly to bovine manure [17,18,20], animal slurry [19], hydrothermally pretreated waste [21], municipal organic solid waste [22], and pretreated barley straw [23]. Nkemka and Muerto [24] used the soluble fraction of seaweed *Ulva* sp. to reduce digestion times and increase methane content in biogas.

Therefore, we asked ourselves if the solid–liquid separation of *Sargassum* spp. biomass increases biodegradability and methane yield, as *Sargassum* spp. biomass contains compounds with complex structures that are difficult to degrade as well as some AD-inhibitory compounds. If *Sargassum* biomass is diluted and separated into its liquid and solid fractions, then it is possible to decrease and distribute such components. Therefore, the novelty of this study is the separation of liquid and solid fractions as a strategy to increase the methane production of *Sargassum* spp., as previous studies had reported a low methane yield. The objectives of this study were (1) to determine the biochemical methane potential and biodegradability index of the biomass fractions and (2) to analyze the composition and distribution of components in the liquid and solid fractions of *Sargassum* spp. biomass.

## 2. Materials and Methods

### 2.1. Biomass and Inoculum Collection

The biomass of *Sargassum* spp. was collected in Playa del Carmen, Q.R., México (20°37′25.1″ N 87°04′23.6″ W). From these samples, two species, *Sargassum fluitans* and *S. natans*, were identified according to morphological characteristics described by Govindarajan et al. [25]. After collection, the samples were transported at room temperature in a cooler to the laboratory where they were stored at −4 °C until use.

The inoculum source was obtained from a pilot-scale fixed-bed reactor located at the Tecnológico Nacional de México, Orizaba campus (18°51′15.9106″ N–97°05′55.8914″ W). This reactor operates at room temperature and processes municipal organic solid waste. The inoculum was then transferred to the laboratory where it was incubated for three weeks at 35 °C and fed with vegetables and fruit waste to ensure methane production and acclimate the microbial population to mesophilic conditions. Before the biochemical methane potential (BMP) test, the inoculum was degassed for 7 days. The inoculum had a pH value of 7.66, and the contents of total solids (TS), volatile solids (VS), ash, total chemical oxygen demand (_T_COD) and soluble chemical oxygen demand (_S_COD) were 3.68 ± 0.10%, 63.86 ± 0.73% ST, 36.14 ± 0.73% ST, 71.36 ± 6.94 g L^−1^, and 3.56 ± 1.10 g L^−1^, respectively.

### 2.2. Biomass Pretreatment and Solid–Liquid Separation

The biomass was rinsed with running water for 2 min to remove sand and other natural contaminants. Part of the biomass was processed to separate a liquid fraction (LF) and a solid fraction (SF), as described below. First, the biomass was diluted with tap water at a 1:1 ratio and ground for 30 s in a 2 HP food processor with a surgical steel blade and Tritan cup, model BLSTXPN7003 (Xpert Series^TM^ Oster, Beijing, China). Next, the liquid fraction was separated, with the aid of a stainless steel multipurpose manual press, using a nylon mesh with a pore size of 150 µm. Finally, the LF was stored at 4 °C until use, whereas the SF was dried at 105 °C for 5 h and reserved in airtight Ziploc^®^ bags (Toluca de Lerdo, Mexico). Another part of the biomass, previously washed and unprocessed (RB), was dried at 105 °C for 5 h and ground in a mortar and pestle to ≤1 mm in size. Finally, the RB was reserved at room temperature in airtight Ziploc^®^ bags until use.

### 2.3. Biochemical Methane Potential (BMP) Tests

The BMP test was performed as described by Holliger et al. [26,27]. The bioreactors, which consisted of serum bottles each with a volume of 120 mL and a working volume of 75 mL, were filled with the RB and SF. Due to the low VS content of the FL, it was necessary to use serum bottles each with a volume of 473 mL and a working volume of 300 mL. Bioreactors were set up with only inoculum and no substrate as blanks (negative control). The inoculum-to-substrate ratio was 2:1 based on the VS content and was calculated using the web application called OBA (Online Biogas App, https://biotransformers.shinyapps.io/oba1/ (accessed on 7 October 2021). The pH values of the cultures were adjusted to 7.2 ± 0.1 at the beginning of the experiment. Subsequently, all serum bottles were hermetically sealed with butyl rubber stoppers and aluminum caps. Finally, the headspace was flushed with 99.5–100% nitrogen gas (Praxair México S. de R.L. de C.V.) for 3 min to attain anaerobic conditions. The bioreactors were incubated at 35 °C until the daily methane production for three consecutive days was <1% of the accumulated volume. Throughout this period, the bottles were shaken manually daily for 60 s to ensure proper mixing and digestion of the media. The biogas volume was measured using 5–60 mL glass syringes with a valve and luer lock system by injecting the needle through the stopper at regular intervals until the biogas production stopped. The methane produced by the bioreactors with the substrate was corrected with the biogas produced by the blanks, and the results were presented as the volume of gas (L) at standard conditions (273 K and 1 atm) by the mass (kg) of VS added. This experiment was conducted in triplicate (*n* = 3).

### 2.4. Analytical Methods

The biogas composition was analyzed in a gas chromatograph (Buck Scientific 310, Norwalk, CT, USA) with a thermal conductivity detector (TCD) equipped with a CTR-I column 6 inches long and 0.25 inches in diameter. Helium was used as carrier gas at 70 psi; the column temperature was 36 °C and the detector temperature was 121 °C. The total solids (TS) and moisture content were determined by drying the samples at 105 °C for 24 h, whereas the ash and volatile solids (VS) content were determined using the muffle furnace at 550 °C for 2 h according to standard methods [28]. Total chemical oxygen demand (_T_COD) and soluble chemical oxygen demand (_S_COD) for the inoculum and the LF were determined with the colorimetric method [28] by using a spectrophotometer (Hach DR/2400, Ames, IA, USA). The pH was determined with a pH meter (Thermo Scientific™ Orion™ Versa star, Waltham, MA, USA).

Total phenol content was determined with the Folin-Ciocalteu method [29] by using a UV-Vis spectrophotometer (Shimadzu, UV 1280, Kioto, Japan). All of the above analyses were performed in triplicate. Elemental composition analysis for the carbon (C), hydrogen (H), and nitrogen (N) content of the raw biomass, LF, and SF samples was performed using an elemental analyzer (PerkinElmer Series II CHNS/O Analyzer 2400, Waltham, MA, USA). Oxygen (O) content was estimated by difference according to the formula given by Tedesco and Daniels [30]. Total sulfur (S) was quantified by turbidimetry with gum arabic as a stabilizer and use of a UV/Vis spectrophotometer (Thermo Scientific™ Spectronic 200, Waltham, MA, USA).

Fiber analysis to determine cellulose, hemicellulose, and lignin content was carried out according to the method proposed by Van Soest et al. [31] in a fiber analyzer ANKOM 200 (ANKOM Technology, Fairport, NY, USA). The content of the main mineral salts was determined using a flame photometer (Sherwood Scientific Ltd., Corning 410, Cambridge, UK) for sodium (Na) and potassium (K), and using an atomic absorption spectrometer (Agilent Technologies, Inc., Varian 240FS, Santa Clara, USA) for calcium (Ca) and magnesium (Mg). All of the previously described analyses were performed in duplicate.

### 2.5. Mass Balance and Stoichiometric Calculations

A mass balance was performed for the solid–liquid separation process based on 1 kg of wet basis biomass. The amounts of the components (i.e., TS, VS, ash, C, N, phenols, and fibers) in each fraction (solid or liquid) after the separation process were calculated using Equation (1):

(1) $$C_{F}\left(g\;kg^{-1}\right)=F\;Mass\;\left(g\right)\times \frac{PC\left(\%\right)}{100}$$

where *C_F_* corresponds to the amount of the component in the fraction per kg of biomass, *F Mass* is the amount of mass of the fraction, and *PC* is the percentage of the component in the fraction on a wet basis. The balance or distribution of the components was calculated using Equation (2):

(2) $$P_{F}\left(\%\right)=\frac{C\;Mass_{F1}}{C\;Mass_{F1}+C\;Mass_{F2}}\times 100\%$$

where *P_F_* is the ratio of the fraction component (solid/liquid), *C Mass_F_*_1_ is the mass of fraction component 1 (solid/liquid), and *C Mass_F_*_2_ is the mass of fraction component 2 (solid/liquid).

The empirical formula (C_n_H_a_O_b_N_c_S_d_) was determined based on the elemental chemical composition of the organic matter present in the pretreated and untreated biomass of *Sargassum* spp. The Theoretical Biochemical Methane Potential (TBMP) was calculated based on the Buswell equation (Equation (3)) and the Boyle equation (Equation (4)) [32]:

(3) $$C_{n}H_{a}O_{b}N_{c}S_{d}+\left(n-\frac{a}{4}-\frac{b}{2}+\frac{3c}{4}+\frac{d}{2}\right)H_{2}O\overset{yields}{\to }\left(\frac{n}{2}+\frac{a}{8}-\frac{b}{4}-\frac{3c}{8}+\frac{d}{4}\right)CH_{4}+\left(\frac{n}{2}-\frac{a}{8}+\frac{b}{4}+\frac{3c}{8}+\frac{d}{4}\right)CO_{2}+cNH_{3}+dH_{2}S$$

(4) $$TBMP\;\left(L\;CH_{4\;}kg\;VS^{-1}\right)=\frac{22.4\times \left(\frac{n}{2}+\frac{a}{8}-\frac{b}{4}-\frac{3c}{8}-\frac{d}{4}\right)}{12n+a+16b+14c+32d}$$

where 22.4 is the volume (*L*) of 1 mole of gas at standard temperature and pressure.

The biodegradability index (*BI*) (Equation (5)) was calculated to estimate the digestion efficiency by biochemical methane potential (*BMP*) assays. The *BI* was calculated as the percentage of the *TBMP* of Equation (4) reached by the substrate at the end of the digestion period [33]. The equation is as follows:

(5) $$BI\;\left(\%\right)=\frac{BMP}{TBMP}\times 100$$

### 2.6. Data Analysis

The kinetic behavior could be described by employing the first-order and modified Gompertz models (Equations (6) and (7)). The two models were used due to the better fit of the models to the net cumulative CH_4_ production results. IBM SPSS version 27 was used to calculate the kinetic parameters using nonlinear least squares regression analysis. The equations are as follows:

(6) $$BMP\;\left(t\right)=BMP_{max}\times (1-exp^{-k\times t})$$

(7) $$BMP\;\left(t\right)=BMP_{max}\times exp\left\{-exp\left[\frac{R_{max}\times e}{R_{max}}\left(\lambda -t\right)+1\right]\right\}$$

where *BMP* is the net cumulative CH_4_ yield (L CH_4_ kg^−1^ VS) at time *t* (day), *BMP_max_* is the maximum CH_4_ potential (L CH_4_ kg^−1^ VS), *k* is the decay constant (day^−1^) which represents the degradation rate of the substrates, *R_max_* is the maximum CH_4_ production rate (L CH_4_ kg^−1^ VS day^−1^), *e* is 2.71828, and *λ* is the lag phase (days) or the number of days before significant CH_4_ production starts.

A one-way analysis of variance (ANOVA) was performed to evaluate the effect of substrate (RB, LF, and SF) on *BMP* with a 95% confidence interval limit. If *p* values were less than 0.05, then the data were considered statistically significant, and a Tukey HSD test (α = 0.05) would then be performed. All statistical analyses were performed with the R program using RStudio (versión 1.3.1093).

## 3. Results and Discussion

### 3.1. Compositions of Raw and Processed Biomass

The results of the physicochemical analysis show a significant difference in the TS, VS, and ash contents of the LF: they were statistically lower (*p* < 0.05) than those of the RB and the SF (Table 1). The TS content in the liquid fraction was significantly low (<1%) due to the solid–liquid separation process that retained most of the solids in the SF. Ash content constituted 32% of the dry weight in the RB and SF, and 40% in the LF. The ash content in seaweed varies widely from 3.5 to 45% [9], whereas in brown seaweed species it can vary from 33 to 55% [34]. Based on the VS/TS ratio values of the substrates, the LF, RB, and SF contained more than 50% organic matter by dry weight. According to Wang et al. [35], a VS content higher than 50% is more suitable for anaerobic digestion. The COD analysis inthe LF showed that more than 60% of the organic matter was in the form of soluble compounds, and about 40% was in particulate form. These characteristics of the LF could reduce the retention times during AD.

The organic elemental analysis (Table 2) shows that the C: N ratio in the SF (18.6) and the RB (19.5) did not present significant differences in their values (*p* < 0.05). Similar C: N ratios ranging from 16 to 22 have been reported with mixed *Sargassum* species [12,14]. It has been described that the optimal C: N ratio for anaerobic digestion is 20–30 [36], as low values may produce lower methane yields due to ammonia inhibition by the degradation of organic nitrogen compounds [37]. However, the C: N ratio for the LF had a value of nine because C decreased by 23% and N increased by 67%. Habig et al. [38] found that some algal species with C: N ratios that were 6–9 showed similar performance to those with a value >19. Therefore, the low C: N ratio in the LF of *Sargassum* spp. does not seem to be a limiting factor for DA. Furthermore, the sulfur content in the *Sargassum* samples was <1%, as has been reported in previous studies with the *Sargassum* spp. [12,14]. This is advantageous for AD, as a high content of sulfur compounds can affect degradation or form H_2_S, which can inhibit methane production [9].

Insoluble fiber content showed a statistically significant difference (*p* < 0.05) between the SF and LF (Table 3). It was observed that the LF presented a higher percentage of insoluble fiber (42.5%) than the SF (23.87%) and RB (32%). The LF was found to have high hemicellulose (16.64%) and lignin (21.4%) content. This result indicates that most of the solids that seeped into the LF were insoluble fibers. These may affect the biodegradability of LFs, as the presence of complex structures creates recalcitrance in the DA [39]. A significant reduction in the content of total phenols in both fractions was also found (*p* < 0.05). The results are expressed in mg gallic acid equivalents (GAE) per unit mass for the SF and per unit volume for the LF. The LF had a reduction corresponding to 96% with respect to the RB. This result could benefit the microbial degradation of *Sargassum* spp. biomass because phenolic compounds can cause low methane yields in brown seaweed, cause cell damage to microorganisms, and inactivate enzymatic activity [37,40]. It has also been reported that the presence of phenols makes brown algae more stable and therefore more resistant to degradation [9].

The inorganic elemental analysis (Table 4) revealed a significant change (*p* < 0.05) in the mineral content of the SF and LF. The RB presented a total mineral content of 116 300 mg kg^−1^, similar to the amount reported by Thompson et al. [14] who found 121 00 mg kg^−1^ in pelagic *Sargassum*. Both the RB and SF had high Ca content, which has also been reported by other authors to be the most abundant in the *Sargassum* spp. [2,5,12]. Surprisingly, after the solid–liquid separation, the total number of minerals decreased in the LF and increased in the SF significantly (*p* < 0.01). Thus, the LF may present advantages in AD because the low methane yields have been attributed to salinity, mainly light metal ions such as Na, K, Ca, and Mg [41]. High levels of salts can generate osmotic pressure and lead to dehydration of methanogenic microorganisms [37].

### 3.2. Production of Biogas and Methane from Biomass

**Figure 1:**
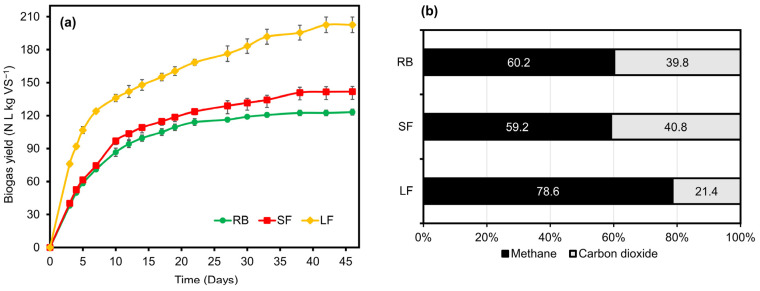
The yield of biogas (**a**) and biogas composition (**b**) of raw biomass (RB), solid fractions (SF), and liquid fractions (LF) of *Sargassum* spp. from the Mexican Caribbean (*n* = 3, error bars are standard deviation).

The biogas yields and their composition after the BMP tests at 46 days are illustrated in Figure 1. As can be observed in Figure 1a, the LF presented the highest biogas yield, with a value of 202.59 ± 8.6 N L Kg VS^−1^, whereas the SF and RB achieved a biogas yield of 141.8 ± 6.4 N L kg VS^−1^ and 123.16 ± 4.35 N L kg VS^−1^, respectively. The composition of the biogas obtained with each substrate in terms of CH_4_ and CO_2_ is shown in Figure 1b. In both the RB and SF, about 60% of the gas was methane. Surprisingly, the LF presented biogas production with methane content of more than 70%, which increases the energy content of the biogas and is beneficial if the biogas needs to be refined to the same level as that of natural gas. The biogas yields and their composition after the BMP tests at 46 days are illustrated in Figure 1.

**Figure 2:**
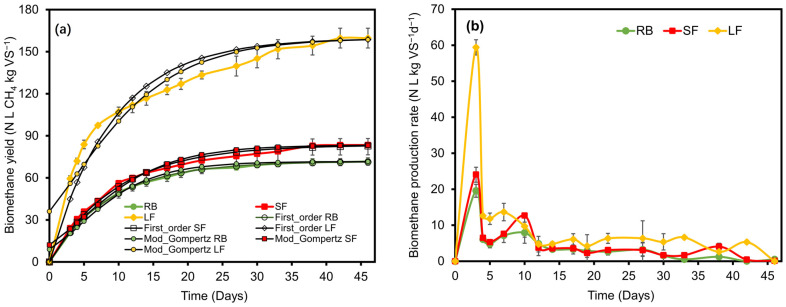
Biomethane yield (**a**) and biomethane production rate (**b**) of raw biomass (RB), solid fractions (SF), and liquid fractions (LF) of *Sargassum* spp. from the Mexican Caribbean. (*n* = 3, error bars are standard deviation).

In terms of methane production, a one-way analysis of variance showed that BMP was very significantly affected by substrate (*p* < 0.001). Figure 2a shows the model results comparing the predicted cumulative methane values with the experimental values. The LF achieved the highest methane yield with a value of 159.7 ± 7.1 N L CH_4_ kg VS^−1^ (Figure 2a). Methane yields from the RB and SF were 83.45 ± 4.6 and 71.7 ± 2.6 N L CH_4_ kg VS^−1^, respectively. The methane yield of the LF was higher than those presented by other authors who used *Sargassum* spp. in solid or semi-solid form, and their results ranged between 41 and 145 L CH_4_ kg^−1^ VS [12,13,14]. Specific methane production curves indicated that the LF had a higher number of available compounds that were easy to degrade and were consumed in the first 10 days. The daily methane production rate by substrates (Figure 2b) showed that the LF presented the highest daily methane production within the first 5 days, corresponding to 37% of the accumulated total. However, the SF and RB presented a slow methane production rate and a longer stationary phase. Tukey’s test showed that the methane yield of the RB and SF were statistically similar (*p* = 0.156).

The kinetic parameters were calculated for each type of substrate (RB, SF, and LF) and are shown in Table 5. An *R*^2^ value closer to one (indicating the fit of the model) was found when using the respective models for the net cumulative CH_4_ production from the RB and SF. This indicates a good fit of the model to the net cumulative CH_4_ production (*R*^2^ > 0.95). Except for the LF, *R*^2^ values were from 0.921 to 0.942. The *k* values varied between 0.104, 0.110, and 0.115 (day^−1^) for the SF, LF, and RB, respectively. The higher *k* values indicate a shorter degradation time, and *k* can display a huge variability which can be specific to a particular process system [42]. Thus, the SF had the lowest degradation rate. The maximum predictable biomethane potential (*BMP_max_*) of the seaweed is shown in Table 5. It can be observed that the LF had the maximum methane yield, followed by the SF. The maximum production rate predicted (*R_max_*) suggested that the LF had the highest value, followed by the RB and SF in descending order. The latency phase was observed to be negative in all cases, which is probably because the gas production profiles do not exhibit lag at all. These results can be observed in Figure 2 where it is not possible to appreciate a lag phase.

### 3.3. Balance and Distribution of the Components of the Solid–Liquid Separation Process

**Figure 3:**
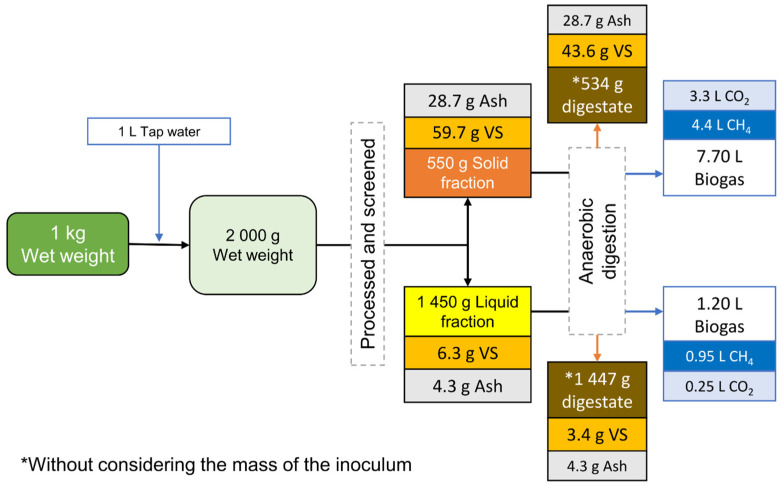
Mass balance based on the content of VS, ash, and amount of biogas from 1 kg of biomass after processing, filtration, and anaerobic digestion.

The mass balance and methane generation after the solid–liquid separation process of 1 kg of wet *Sargassum* spp. biomass is illustrated in Figure 3. It was observed that the highest concentration of solids (VS and ash) was retained in the SF, and this amount was eight times the concentration of solids present in the LF. The amount of biogas (CH_4_ and CO_2_) produced by the SF was 6.4 times greater than the amount generated by the LF. This is because the SF retains a greater amount of VS; therefore, there was less substrate available for biogas generation. Similar results were reported with cattle manure after a coagulation–flocculation and sieving process: the solid fractions retained the highest amount of VS and therefore generated a higher amount of methane [17,20].

The results of the component distribution of the solid–liquid separation process of biomass are shown in Table 6. It can be observed that 26.6% of the initial mass was the SF and 73.4% was the LF. It was found that the amounts of TS and VS retained in the SF were 89.2% and 90.5%, respectively. The high VS content in the SF generated the highest amount of methane (82.2%) from 1 kg of wet biomass. C content was retained at 90.1% in the SF whereas N content was retained at only 81.6%, indicating a slight migration of nitrogenous compounds into the LF, thus causing a low C: N ratio.

The solid–liquid separation generated a change in the distribution of the lignocellulosic components. The SF retained 95.6% of the cellulose content and 80.3% of the lignin content. However, the hemicellulose content was distributed toward the LF (55.3%). Similar results where high amounts of hemicellulose and lignin are retained in the liquid fraction after pretreatment have been reported [43]. It is necessary to improve the solid–liquid separation process to retain even more insoluble fibers because, as described in Section 3.1, the LF presented more than 40% of fibers.

Surprisingly, although the concentration of total phenols in the LF was low, the highest concentration of phenols was distributed toward the LF, where 69.5% of the total was accumulated. This is because a higher amount of the LF was obtained, and also because some phenolic compounds are soluble in water.

### 3.4. Efficiency of Anaerobic Digestion and Energy Evaluation

The summary of the AD process after 46 days (Table 7) showed that the LF presented the highest methane yield with a biodegradability index (BI) of 39%. The BI varies according to the seaweed species (19–85%) [44]. Milledge et al. [12] reached an IB of 17–37% similar to this study but using the *Sargassum* species separately. Possible reasons why the LF did not reach a higher BI include the high content of total phenols, the low C: N ratio, or the concentration of insoluble fibers, mainly hemicellulose and lignin. It is necessary to improve the solid–liquid separation process to increase the retention of recalcitrant compounds in the SF and the recovery of soluble compounds in the LF.

The AD had good _S_COD (97.4%) and _T_COD (83.8%) removal in the LF. The _T_COD removal was lower than _S_COD, indicating that some solids were not degraded, such as some fibers. This result is important because, on a large scale, the effluent could be discharged freely to comply with current environmental regulations. Additionally, the low-COD effluent could be recirculated for use as dilution water before processing and solid–liquid separation.

In the case of the SF, although it retained a large amount of organic matter, it presented a low BI (24.8%). Therefore, pretreatments focused on improving the hydrolysis of the SF are required.

Energy evaluation was performed and calculated as described by Montingelli et al. [45]. After AD, it was found that the energy produced by the LF biogas was higher than the energy produced by the SF and RB. However, the net energy produced from the LF is lower than the net energy produced by the SF and RB because more energy is required to obtain 1 g of VS from the LF, as shown in Table 6. Considering the net energy produced from the LF and SF, a positive energy gain of 45.94% was obtained when processing the biomass. The solid and liquid fractions provide a positive energy gain of 1.5 times greater than using raw biomass.

Therefore, the solid–liquid separation of *Sargassum* spp. biomass has the advantage of producing a liquid fraction with better characteristics for AD. The characteristics of the LF of *Sargassum* spp. make it suitable for treatment in high-load anaerobic reactors with shorter hydraulic retention times using smaller reactors with higher methane production rates than conventional anaerobic reactors [20].

Pilot testing of the liquid fraction in high-load reactors such as those used in wastewater treatment should be considered before the scale-up of this system. A techno-economic analysis should be considered to know if methane production compensates for the use of freshwater used in solid–liquid separation, as this is a valuable resource.

## 4. Conclusions

In this study, the solid–liquid separation succeeded in separating and retaining the largest number of complex compounds difficult to degrade in the solid fraction, which generated a liquid fraction with better biodegradability and better methane yield than the raw biomass. The liquid fraction reached a biodegradability index of 39% with a methane yield higher than that achieved with the solid fraction and the raw biomass. In addition, the biogas presented a methane content higher than 78%. The characteristics of the liquid fraction would allow its treatment in large-scale high-load reactors. It is still unclear why the liquid fraction did not reach a higher biodegradability index, but it is possibly due to its high concentration of phenols, hemicellulose, and its low C: N ratio. Therefore, the phenol concentration in the biomass prior to solid–liquid separation should be considered. The solid fraction had low biodegradability and a methane yield statistically equal to that of the raw biomass. Considering that the solid fraction retains a large amount of organic matter, other attractive options for use could be explored, such as composting for use as a soil conditioner. The authors suggest that that with improvement of the screening process and with the pretreatment of biomass before the solid–liquid separation, the retention of insoluble compounds and the recovery of soluble compounds could be improved.

## Figures and Tables

**Table 1:** Physicochemical analysis of raw (RB) and processed *Sargassum* spp. biomass from the Mexican Caribbean: liquid (LF) and solid fractions (SF) on a wet basis (*n* = 3). Means with different superscripts in rows indicate statistically significant differences between samples (*p* < 0.05).

	RB	Processed Biomass	
		LF	SF
Moisture (%)	80.3 ± 0.95 ^b^	99.3 ± 0.03 ^a^	83.9 ± 0.22 ^b^
TS (%)	19.7 ± 0.95 ^b^	0.70 ± 0.08 ^a^	16.1 ± 0.22 ^b^
SV (%)	13.2 ± 0.8 ^b^	0.42 ± 0.04 ^a^	10.8 ± 0.20 ^b^
Ash (%)	6.4 ± 0.12 ^b^	0.28 ± 0.03 ^a^	5.2 ± 0.07 ^b^
VS/TS	0.67 ± 0.01 ^b^	0.59 ± 0.01 ^a^	0.67 ± 0.01 ^b^
_T_COD (g L^−1^)	NA	10.4 ± 0.53	NA
_S_COD (g L^−1^)	NA	6.26 ± 2.01	NA
EC (mS cm^−1^)	NA	1.77	NA
TDS (mg L^−1^)	NA	1132.8	NA
pH @ 27 °C	NA	7.10	NA

EC = Electrical conductivity. TDS = Total dissolved solids = EC (dS m^−1^) × 640. NA = Not analyzed.

**Table 2:** The organic elemental analysis of raw (RB) and processed *Sargassum* spp. biomass from the Mexican Caribbean: liquid (LF) and solid fractions (SF) on a dry basis (*n* = 1).

	RB	Processed Biomass	
		LF	SF
C (%)	35.5	27.3	29.8
H (%)	3.83	3.50	2.67
O (%)	25.6	25.6	32.9
N (%)	1.82	2.99	1.60
S (%)	0.34	0.12	0.36
C: N Ratio	19.5	9.0	18.6
Empirical formula ^a^	C_3_H_3.8_O_1.6_N_0.12_	C_2.3_H_3.4_O_1.6_N_0.21_	C_2.5_H_2_._6_O_2.0_N_0.11_

^a^ The sulfur content is negligible in the empirical formula due to the low value of the sulfur content (0.01).

**Table 3:** Structural analysis of raw (RB) and processed *Sargassum* spp. biomass from the Mexican Caribbean: liquid (LF) and solid fraction (SF) on a dry basis (*n* = 2).

	RB	Processed Biomass	
		LF	SF
Insoluble fiber (%)	32.0 ± 0.9	42.5 ± 0.2	23.87 ± 1.1
Cellulose (%)	14.8 ± 0.7	4.5 ± 0.1	11.7 ± 0.7
Hemicellulose (%)	0.47 ± 0.3	16.64 ± 0.1	1.62 ± 0.9
Lignin (%)	16.8 ± 0.9	21.4 ± 0.6	10.52 ± 0.9
Total phenols ^a^	14.4 ± 0.8	0.53 ± 0.04 ^b^	4.05 ± 0.5

^a^ mg GAE g^−1^. ^b^ mg GAE mL^−1^.

**Table 4:** The inorganic elemental analysis of raw (RB) and processed *Sargassum* spp. biomass from the Mexican Caribbean: liquid (LF) and solid fractions (SF) on a dry basis (*n* = 1).

	RB	Processed Biomass	
		LF	SF
Na (mg kg^−1^)	7900.0	4900.0 ^b^	4400.0
K (mg kg^−1^)	7300.0	70.0 ^b^	1800.0
Ca (mg kg^−1^)	93,000.0	900.0 ^b^	150,600.0
Mg (mg kg^−1^)	8100.0	170.0 ^b^	11,800.0
Total minerals	116,300.0 ^a^	6040.0 ^b^	168,600.0 ^a^

^a^ mg kg^−1^. ^b^ mg L^−1^.

**Table 5:** Results of kinetics study (first-order and modified Gompertz).

	First-Order			Modified Gompertz			
	*BMP_max_* (L CH_4_ kg VS^−1^)	*K* (Day^−1^)	*R* ^2^	*BMP_max_* (L CH_4_ kg VS^−1^)	*R_max_* (L CH_4_ kg VS^−1^ Day^−1^)	*λ* (Day)	*R* ^2^
RB	71.30	0.115	0.999	71.59	4.384	−1.609	0.982
SF	82.75	0.104	0.994	83.22	4.332	−2.432	0.970
LF	158.66	0.110	0.942	158.57	6.853	−5.165	0.921

**Table 6:** Distribution of biomass components after the solid–liquid separation process.

	Raw Biomass (g kg^−1^)	Solid Fraction (g kg^−1^)	Distribution (%)	Liquid Fraction (g kg^−1^)	Distribution (%)
TS	196.5	88.65	89.2	10.69	10.8
VS	132.0	59.73	90.5	6.26	9.5
Ash	64.0	28.70	87.04	4.30	12.96
Carbon	69.8	26.42	90.1	2.92	9.9
Nitrogen	3.60	1.42	81.6	0.32	18.4
Cellulose	29.08	10.4	95.6	0.5	4.4
Hemicellulose	0.92	1.44	44.67	1.78	55.33
Lignin	33.01	9.33	80.30	2.30	19.72
Phenols	2.83	0.35	30.5	0.81	69.5
Methane ^a^	8.84	4.40	82.2	0.95	17.8
Mass	1 kg	0.55 kg	26.6	1.45 kg	73.4

^a^ L kg biomass^−1^.

**Table 7:** Evaluation of anaerobic digestion: biodegradability index, organic matter removal percentages, and energy recovery of raw biomass (RB), solid fractions (SF), and liquid fractions (LF). Means with different superscripts indicate statistically significant differences between samples (*p* < 0.05).

	Biomethane Potential (L CH_4_ kg^−1^ VS)		Efficiencies (%)				Energy Recovery (Wh g^−1^ VS)		
	Experimental	Theoretical	BI	VS Removal	_T_COD Removal	_S_COD Removal	E_p_	E_c_	Net E_p_
RB	71.7 ± 2.6 ^b^	501.51	14.3	15.1	NA	NA	0.69	0	0.69
SF	83.45 ± 4.6 ^b^	336.60	24.8	27.0	NA	NA	0.76	0.12	0.64
LF	159.7 ± 7.1 ^a^	409.78	39.0	45.5	84.0	90.0	1.53	1.16	0.38

NA = Not analyzed. E_p_ = Energy produced. E_c_ = Energy consumed.

## Data Availability

All data are available in the manuscript.
